# Benzocaine-Induced Methemoglobinemia in a Postoperative Bariatric Patient following Esophagogastroduodenoscopy

**DOI:** 10.1155/2019/1571423

**Published:** 2019-02-24

**Authors:** Khuram Khan, Irwin White-Gittens, Saqib Saeed, Leaque Ahmed

**Affiliations:** Department of Surgery, Harlem Hospital Center, Columbia University Medical Center, New York, NY, USA

## Abstract

Methemoglobinemia is a rare complication in clinical practice. It is most commonly seen in patients undergoing endoscopic procedures, including EGDs, laryngoscopies, bronchoscopies, and nasogastric tube insertions. This is thought to be a disease seen almost exclusively in patients with genetic predispositions to develop it; the increasing use of topical anesthetics during procedures has made methemoglobinemia a disease entity that every clinical provider should be able to recognize and treat. Clinically, patients become cyanotic with mild oxygen derangements on pulse oximetry, in the range of 84 to 90%. Paradoxically, these patients demonstrate normal to supranormal oxygen levels in the blood on blood gas analysis. We report a case of 34-year-old female postoperative Roux-en-Y gastric bypass patient who developed hypoxia and cyanosis after a routine EGD procedure to relieve a food impaction. Differentials of aspiration and pulmonary embolism were plausible; stat blood gas analysis clinched the diagnosis and managed with intravenous methylene blue.

## 1. Introduction

Causes of methemoglobinemia include topical anesthetics (mainly esters), dapsone, nitrates (e.g., nitroglycerin), and metoclopramide [1]. The mechanism by which methemoglobinemia develops is due to an altered state of the hemoglobin tetramer. When methemoglobin is in the altered state or oxidized state, it results in decreased affinity for oxygen resulting in decreased oxygen delivery to tissues, which adversely affects oxygen transport to the tissues. When methemoglobin levels in the blood rise above a certain level, cyanosis develops. Higher levels of methemoglobin in blood could have significant cardiac and cerebral morbidity and mortality.

## 2. Case Presentation

A 34-year-old female, with preoperative BMI of 42.8, status postlaparoscopic Roux-en-Y gastric bypass (LRYGB) with normal postoperative upper gastrointestinal series three weeks prior, presented with a two-hour history of severe epigastric and upper abdominal pain after ingesting a large piece of meat. Vital signs were stable. On physical examination, she was tender in the upper abdomen without peritoneal signs. The rest of her exam was unremarkable. White blood cell count was elevated to 15.3K with otherwise normal labs. Differential diagnosis of food impaction was made and EGD was recommended. Interestingly, the patient had an uneventful EGD prior to her surgery. Before IV sedation was administered, the patient reported that she was uncomfortable and felt the endoscope in the back of her throat during the last procedure, and so she received 5 sprays of 20% benzocaine prior to intubation. The EGD demonstrated an empty stomach and a relatively tight gastrojejunostomy with stigmata of recent food impaction, including erythema, excoriations, and mild edema. There were no signs of aspiration at the end of the procedure, but she was noted to be extremely drowsy despite only receiving 50mg Fentanyl and 2mg Versed. As such, attempts were made to rapidly reverse these medications with Naloxone and Flumazenil before transferring her to the postanesthesia care unit (PACU), while in PACU she was initially oriented but suddenly became obtunded, then unconscious, cyanotic, and desaturating to mid-1980s. Nonrebreather mask oxygen did not improve her saturation. Given this, she was intubated and an arterial blood gas was drawn. During the blood draw, the blood was noted to be dark brown to black in color. The results of the ABG were as follows: pH, 7.43; PaO2, 217 mm Hg; PaCO2, 28.1 mm Hg; and methemoglobin, >30%. Cyanosis in the setting of recent topical anesthetic use, along with her ABG results, gears toward the diagnosis of methemoglobinemia and 100 mg of 1% methylene blue was given intravenously over 30 minutes with incremental improvement of her respiratory parameters and cyanosis. She was subsequently transferred to the intensive care unit. Repeated ABG showed a methemoglobin concentration of 5.5% after one hour. Patient had successive ABG analyses over the next couple of hours showing progressive decrease in methemoglobin levels: 30%, 5.5%, and 2.4%. She fully recovered two hours after onset. She was extubated the following day and was discharged home on the fourth post-EGD day. One week later she was seen in clinic for follow-up and was doing well.

## 3. Discussion

Methemoglobinemia is a disease that even the most experienced clinicians can miss. This is mainly because the presentation may be subtle and/or patients who develop it may have several other disease entities/conditions that can confound the diagnosis. Methemoglobinemia can be either congenital or acquired. The latter is more common as mentioned before which is mainly seen following administration of topical anesthetics for patient comfort prior to laryngeal intubation; the medication is rapidly absorbed and can have its desired effects within seconds and adverse effects within minutes. In addition to this, amide type-local anesthetic produces o-toluidine that is also known to induce Met-Hb formation as well. In a study, benzocaine, an ester, was the mostly commonly implicated medication [2]. It was also described in patients taking dapsone and nitrates such as nitroglycerin among others. In the congenital form, cytochrome B5 reductase enzyme deficiency and hemoglobin M disease are usually implicated [3].

Methemoglobin formation occurs naturally in red blood cells of all mammals [3]. As reported in a study the incidence of methemoglobinemia after performing over 28,000 EGDs is 0.067% [2]. It is estimated that 0.3-0.7% of hemoglobin is oxidized to the methemoglobin molecule daily. In humans, there are several intrinsic mechanisms used to reduce the methemoglobin molecule to its natural form.

Hemoglobin is a specialized protein molecule that transports oxygen from the lungs to tissues. When red blood cells transport oxygen to tissues with low partial pressures of oxygen, it releases the oxygen in its molecular form (O_2_), reducing iron back to its Fe^2+^ form. However, a small percentage of oxygen holds on to an extra electron, creating a superoxide (O^−^) and reducing iron further to its ferric (Fe^3+^) form, also known as methemoglobin. This ferric form of iron cannot bind oxygen, and this new configuration causes a conformation change in the hemoglobin molecule, resulting in increased affinity of the already bound O_2_ on the other iron molecules [4]. This shifts the oxygen dissociation curve to the left, decreasing the ability to offload oxygen to the tissues. This explains why, although the dissolved oxygen (PaO_2_) may be normal or high, the total oxygen bound to the hemoglobin molecule, when methemoglobin which develops will be low, resulting in cyanosis. Therefore, medications that can oxidize ferrous iron to its ferric form and superoxide produce exponentially more methemoglobin, overwhelming the ability of the body to reduce it.

Patients who develop cyanosis in the hospital setting that does not respond to supplemental oxygen, methemoglobinemia, should be suspected. Intubation may be necessary if severe and an ABG will aid in the diagnosis with elevated methemoglobin levels above 10%. Intravenous methylene blue at 1-2mg/kg is given over a five- to thirty-minute period [5], if necessary; it is repeated if levels remain elevated >20%. This works by reducing the methemoglobin to its ferrous iron form, restoring the oxygen carrying capacity of hemoglobin. In our patient, she most likely received a higher than normal dose of the medication.

## 4. Conclusion

Methemoglobinemia is likely an underdiagnosed clinical entity. It is most commonly seen with topical anesthetics administered for patient comfort prior to invasive procedures, resulting in varying degrees of cyanosis and hypoxia. If clinically significant, the majority of patients can be managed successfully with oxygen supplementation and IV methylene blue with favorable outcomes.

## Data Availability

Data sharing is not applicable to this article as no datasets were generated or analyzed during the current study.

## Abbreviations

LRYGB: Laparoscopic Roux-en-Y gastric bypass
EGD: Esophagogastroduodenoscopy
ABG: Arterial blood gas.
